# Effects of tDCS during inhibitory control training on performance and PTSD, aggression and anxiety symptoms: a randomized-controlled trial in a military sample

**DOI:** 10.1017/S0033291721000817

**Published:** 2022-12

**Authors:** Fenne M. Smits, Elbert Geuze, Dennis J. L. G. Schutter, Jack van Honk, Thomas E. Gladwin

**Affiliations:** 1 Brain Research & Innovation Centre, Ministry of Defence, Utrecht, the Netherlands; 2 Department of Psychiatry, UMC Utrecht Brain Center, University Medical Center Utrecht, Utrecht, the Netherlands; 3 Experimental Psychology, Helmholtz Institute, Utrecht University, Utrecht, the Netherlands; 4 Department of Psychiatry and Mental Health, University of Cape Town, Cape Town, South Africa; 5 Institute of Infectious Disease and Molecular Medicine, University of Cape Town, Cape Town, South Africa; 6 Behavioural Science Institute, Radboud University Nijmegen, Nijmegen, The Netherlands; 7 Institute for Lifecourse Development, University of Greenwich, London, UK

**Keywords:** Aggression, anxiety, inferior frontal gyrus, inhibitory control, PTSD, transcranial direct current stimulation

## Abstract

**Background:**

Post-traumatic stress disorder (PTSD), anxiety, and impulsive aggression are linked to transdiagnostic neurocognitive deficits. This includes impaired inhibitory control over inappropriate responses. Prior studies showed that inhibitory control can be improved by modulating the right inferior frontal gyrus (IFG) with transcranial direct current stimulation (tDCS) in combination with inhibitory control training. However, its clinical potential remains unclear. We therefore aimed to replicate a tDCS-enhanced inhibitory control training in a clinical sample and test whether this reduces stress-related mental health symptoms.

**Methods:**

In a preregistered double-blind randomized-controlled trial, 100 active-duty military personnel and post-active veterans with PTSD, anxiety, or impulsive aggression symptoms underwent a 5-session intervention where a stop-signal response inhibition training was combined with anodal tDCS over the right IFG for 20 min at 1.25 mA. Inhibitory control was evaluated with the emotional go/no-go task and implicit association test. Stress-related symptoms were assessed by self-report at baseline, post-intervention, and after 3-months and 1-year follow-ups.

**Results:**

Active relative to sham tDCS neither influenced performance during inhibitory control training nor on assessment tasks, and did also not significantly influence self-reported symptoms of PTSD, anxiety, impulsive aggression, or depression at post-assessment or follow-up.

**Conclusions:**

Our results do not support the idea that anodal tDCS over the right IFG at 1.25 mA enhances response inhibition training in a clinical sample, or that this tDCS-training combination can reduce stress-related symptoms. Applying different tDCS parameters or combining tDCS with more challenging tasks might provide better conditions to modulate cognitive functioning and stress-related symptoms.

## Introduction

Post-traumatic stress disorder (PTSD) and anxiety are mental health disorders that are difficult to treat, particularly among military patients (Spinhoven et al., 2016; Straud, Siev, Messer, & Zalta, 2019). New treatment targets may be provided by finding ways to restore deficits in neurocognitive processes. Across patients with PTSD, anxiety, and impulsive aggression, dysregulated neurocognitive processes center around hyperresponsive limbic regions including the amygdala and (dorsal) anterior cingulate cortex (ACC) (Craske et al., 2017; Davidson, Putnam, & Larson, 2000; Hayes, Hayes, & Mikedis, 2012) and hyporesponsive regions in the lateral and medial prefrontal cortex (PFC), accompanied by impairments in cognitive functions like working memory, cognitive flexibility, and inhibitory control (Etkin, Gyurak, & O'Hara, 2013).

Of these cognitive functions, inhibitory control particularly may play a vital role. Inhibitory control comprises the ability to withhold automatic or context-inappropriate responses in order to maintain goal-directed behavior. PTSD patients display impairments specifically on inhibitory control tasks (DeGutis et al., 2015) and hypoactivation in the brain's hub of inhibitory control: the right inferior frontal gyrus (IFG) (Aron, Robbins, & Poldrack, 2014; Hayes et al., 2012). It is proposed that failing inhibition of inappropriate stress responses, memories, and motor reactions to fear-evoking stimuli contributes to symptoms of hyperarousal and irritability, and in turn, avoidance of fear- or trauma-related triggers and defensive aggression (Aupperle, Melrose, Stein, & Paulus, 2012; van Rooij & Jovanovic, 2019). Moreover, impairments in the prefrontal inhibitory control circuit may impede therapy response (Marwood, Wise, Perkins, & Cleare, 2018). An appealing question is therefore whether the dysregulated inhibitory control circuit poses a potential therapeutic target.

To restore dysregulated brain circuits, transcranial direct current stimulation (tDCS) may play a role by promoting neural plasticity (Yavari, Jamil, Mosayebi Samani, Vidor, & Nitsche, 2018). While tDCS alone may not effectively modulate emotional distress (Smits, Schutter, van Honk, & Geuze, 2020), deficient cognitive processes underlying stress-related disorders – such as inhibitory control – could comprise convenient tDCS targets in this context. For example, single-session tDCS over the right IFG has shown to increase inhibitory control task performance (Mayer et al., 2020; Schroeder, Schwippel, Wolz, & Svaldi, 2020). Inhibitory control can also be enhanced with other techniques used to modulate right IFG functioning (e.g. transcranial magnetic stimulation or neurofeedback by functional magnetic resonance imaging) (Alegria et al., 2017; Zandbelt, Bloemendaal, Hoogendam, Kahn, & Vink, 2013). Interestingly, multiple-session tDCS combined with response inhibition training has demonstrated cumulative effects on inhibitory control performance in healthy volunteers (Ditye, Jacobson, Walsh, & Lavidor, 2012). Increasing evidence now suggests that combining multiple tDCS sessions with cognitive training may produce stronger, more consistent, and longer-lasting effects on and beyond the trained function (Berryhill & Martin, 2018). Combining multiple-session tDCS with inhibitory control training may thus provide opportunities to target impairments in the prefrontal inhibitory control function. The next step in exploring the potential of tDCS-enhanced inhibitory control training in treating stress-related disorders is to replicate these effects in a clinical sample and test whether this beneficially affects clinically relevant outcomes.

In this randomized-controlled trial (RCT), we applied a 5-session inhibitory control training with anodal tDCS over the right IFG in military veterans and active-duty personnel with PTSD, anxiety, or impulsive aggression. As a primary outcome, we tested whether tDCS enhanced inhibitory control during training. As secondary outcomes, we tested tDCS-related changes in inhibitory control performance and stress-related symptoms over the intervention period.

## Methods

This double-blind RCT was preregistered at the Netherlands Trial Register (www.trialregister.nl, ID: NL5709).

### Participants

Military veterans and active-duty personnel of the Dutch Ministry of Defence were recruited between May 2016 and October 2019 through advertisements in mental healthcare outpatient clinics. The following inclusion criteria were applied: 18–60 years of age, fulfilling diagnostic criteria and receiving treatment for PTSD, an anxiety disorder or impulsive aggression problems. Exclusion criteria: primary diagnosis for major depressive disorder (comorbid depression was not a reason for exclusion), substance addiction, severe neurological or psychotic disorder, serious head trauma or surgery, large metal or ferromagnetic parts in the head, implanted pacemaker or neurostimulator, pregnancy, skin damage on the scalp, and neurostimulation in the past month. Psychoactive medication use was assessed. Patients were asked to keep stable doses during the tDCS intervention, starting two weeks in advance. The a priori computed sample size was 96 [48 per group; computed in G*Power 3.1 (Faul, Erdfelder, Lang, and Buchner, 2007) with *α* = 0.05, *β* = 90%, and Cohen's *f* = 0.34 based on results from Ditye and coworkers (Ditye et al., 2012) lowered by 10%]. The medical ethical committee of the University Medical Center Utrecht approved the study. All participants provided written informed consent. The authors assert that all procedures contributing to this work comply with the ethical standards of the relevant national and institutional committees on human experimentation and with the Helsinki Declaration of 1975, as revised in 2008.

### Procedure and randomization

**Fig. 1.**
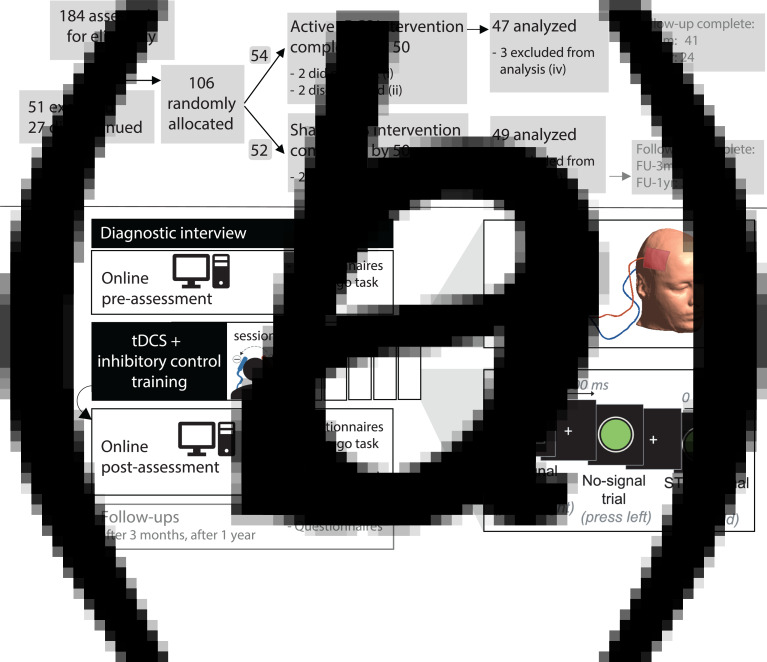
(*a*) CONSORT study flow diagram. FU-3m = 3-months follow-up assessment. FU-1yr = 1-year follow-up assessment. (i) Reasons: delayed discovery of tDCS safety contraindication (*n* = 1), time conflict with other treatment/work (*n* = 1). (ii) Reasons: panic symptoms at tDCS work-up session 1 (*n* = 1), time conflict with other treatment (*n* = 1). (iii) Reasons: time conflict with other treatment/work (*n* = 2). (iv) Reasons: psychoactive drug changes during intervention (*n* = 1), >5 days between tDCS sessions (*n* = 1), tDCS applied at <1.25 mA on request of participant (*n* = 1); (v) Reasons: inadequate performance of the stop-signal task (*n* = 1); (*b*) Overview of study procedure.

Figure 1*b* depicts the study procedure. First, a clinical diagnostic interview was done, including the SCID-I for DSM-IV-R Axis-I disorders (First, Spitzer, Gibbon, & Williams, 2002), DSM-5 intermittent explosive disorder criteria (Coccaro, 2012), and M.I.N.I. ADHD criteria (Sheehan et al., 1998). Patients were then allocated to active or sham tDCS (1:1) by the next available stimulator-activating code from a randomized list (Matlab ‘rand’ function; 20 codes for active tDCS, 20 codes for sham), stratified by eye movement desensitization and reprocessing (EMDR) therapy *v.* cognitive behavioral therapy (CBT) to avoid confounding with psychotherapy effects. Experimenters were blind for code-to-condition correspondence, and, although not formally tested, patients were not expected to know whether they received sham or active tDCS (Ambrus et al., 2012). The interview and tDCS sessions were carried out in test rooms at the University Medical Center Utrecht. Pre- and post-assessments took place online through a weblink.

### tDCS

Participants received five tDCS sessions, with 1–5 days between sessions depending on the participant's availability. TDCS was applied for 20 min over two 5 × 7 cm electrodes by a neuroConn DC-stimulator Plus with settings based on Ditye's study (Ditye et al., 2012): 1.25 mA (fade-in: 8 s), anode on the crossing point between 10-20 system EEG positions T4-Fz and F8-Cz, cathode over the left orbital region (see Fig. 1*b*). Sham tDCS was applied by a 16-s fade-in fade-out stimulation at the start and end of the stimulation period, interleaved by occasional 15 ms pulses of 0.11 mA. The emotional state was assessed before and after each session by the STAI-6 (Marteau & Bekker, 1992), together with possible tDCS side effects scored from 1 (‘absent’) to 4 (‘severe’) (Brunoni et al., 2011).

### Inhibitory control training

TDCS was combined with a 30-min training on the stop-signal task, see Fig. 1*b* (Logan, Cowan, & Davis, 1984). Participants were instructed to quickly press the left or right arrow button upon stimulus presentation (circle or square), but to withhold their response when a stop-signal was heard: an auditory ‘beep’ (25% of trials, 0–400 ms stop-signal onset delay). To titrate successful stop-signal response inhibition to ~50%, stop-signal delays increased or decreased with 50 ms after successful or unsuccessful stopping, respectively. Six blocks of 100 trials were interleaved by 1-min breaks. One extra block with 20 no-signal trials to prevent response slowing was excluded from data analysis. The stop-signal response time (SSRT), the time it takes to stop an already initiated response which reflects inhibitory control, was computed by the independent horse-race model (Logan et al., 1984) and constituted our primary outcome measure. Response speed (RT on no-signal trials) was taken as a control measure.

### Secondary outcome measures of inhibitory control

Prolonged effects of training combined with active *v.* sham tDCS on inhibitory control were tested by comparing performance at pre- *v.* post-assessment on the emotional go/no-go task and the implicit association task (IAT).

The go/no-go task was used to measure the inhibition of prepotent responses driven by a high frequency of go-stimuli. Participants were instructed to rapidly tap on the space bar when a go-stimulus appeared (80% of trials), and to withhold their response to a no-go-stimulus (20% of trials). On 50% of all trials, ‘go’- and ‘no-go’-stimuli (‘[]’ and ‘][’) were superimposed on male face images with a neutral or angry expression [Bochum Emotional Stimulus Set, BESST (Thoma, Soria Bauser, and Suchan, 2013)], to assess threat-related distraction on inhibition performance (Gladwin, Möbius, & Vink, 2019). Stimuli were presented for 600 ms with a 250–350 ms inter-trial interval in 7 blocks of 40 trials. The median reaction time (RT) over go-trials was used to assess effects on response speed, and accuracy represented the ability to correctly execute or inhibit responses. The first (practice) block, the first four trials of each block, post-error trials, sequences of ⩾ 5 consecutive no-response go-trials, and trials with an RT<170 ms were excluded from analysis (on average, 18.5% of trials were excluded).

The IAT was used to measure inhibition of prepotent responses driven by automatic associations. We used the standard IAT with flower and insect names as target words and pleasant and unpleasant words as attributes (Greenwald, McGhee, & Schwartz, 1998). Participants were instructed to classify target and attribute words as quickly as possible by pressing the ‘F’ or ‘J’ button. Each category contained 15 practice trials and 60 test trials. Better inhibition of the automatic response attenuates the increase in response latency and error rate on incongruent trials (the IAT effect). The D600 IAT effect was computed by adding 600 ms to incorrect response RTs, and dividing the difference in congruent *v.* incongruent trial RTs by the RT standard deviation. In addition, a Quad model (Conrey, Sherman, Gawronski, Hugenberg, & Groom, 2005) was estimated based on trial-level classification errors using a multinomial tree processing model in R (Singmann & Kellen, 2013), to quantify the ‘overcoming bias’ (the likelihood that the automatic association is overcome), representing the unique contribution of inhibitory control on IAT performance.

At post-assessment, participants additionally performed a dot-probe task. Unlike the inhibitory control tasks, this task assesses attentional biases for threat. The main outcomes of this task are described in the online Supplementary Materials.

### Symptoms

Beside baseline symptom assessment by the diagnostic interview, symptom levels were assessed at pre-, post-, and follow-up-assessments by self-report scales including the PTSD Checklist for DSM-5 (PCL-5) (Weathers et al., 2013), the trait version of the positive and negative affect schedule (PANAS) (Watson, Clark, & Tellegen, 1988), and the STAXI-2 (Spielberger, 1999). TDCS effects on disorder-specific symptoms of PTSD, anxiety, and impulsive aggression were tested only within subgroups of participants who fulfilled the criteria for the corresponding diagnosis. Depressive symptoms and general mental well-being were assessed using the Beck Depression Inventory 2nd edition (BDI-II) (Beck, Steer, & Brown, 1996) and the Outcome Questionnaire 45 (OQ45) (Lambert, Finch, & Maruish, 2004). At baseline, childhood trauma and impulsivity traits were assessed by the Dutch version of the childhood trauma questionnaire short form (CTQ-SF) (Bernstein et al., 2003) and Barrett's Impulsivity Scale (BIS-11) (Patton, Stanford, & Barratt, 1995).

### Statistical analysis

Continuous outcomes were analyzed in mixed-design ANOVAs in R (R Foundation for Statistical Computing, 2016) with the ‘rstatix’ package (Kassambara, 2020). Trial-level accuracy data were, as recommended (Jaeger, 2008), analyzed in binary logistic mixed-effects models with the ‘lme4’ package (Bates, Mächler, Bolker, & Walker, 2015) with a random intercept for the participant, where *p* values were obtained in likelihood ratio tests of the full model *v.* a model without the effect. *Stimulation group* (active *v.* sham tDCS) was treated as between-subjects factor, *Time* (tDCS sessions 1–5, or pre-assessment, post-assessment and follow-ups) as within-subjects factor, and their interaction would reflect whether the active tDCS intervention induced different time effects than the sham intervention. *Age* and *Use of psychoactive medication* (yes/no) were included as covariates. Where the assumption of sphericity was violated, Greenhouse-Geisser-corrected results are reported. Effects are reported as significant at *p* < 0.05. Effect sizes are reported as generalized eta-squared (*η*^2^_G_).

Additionally, to provide possibly useful information for neurocognitive models about the relationship between inhibitory control and stress-related symptoms, we computed baseline correlations between the inhibitory control tasks and symptom scores at pre-assessment. Also, to explore if improved inhibitory control could drive symptom relief, we tested in a regression model if (i) SSRT improvement (ΔSSRT = SSRT session 5 − SSRT session 1) or (ii) the achieved SSRT level on session 5 predicted reductions in PTSD, anxiety, or anger symptoms (Δsymptom score = post-score − pre-score). Here, *Stimulation group* was always entered as a first predictor to control for effects attributable to tDCS.

## Results

Figure 1*a* shows the study flow. As can be seen in Table 1, the active tDCS and sham groups matched on most factors. Yet, despite random group allocation, females and post-active veterans were overrepresented in the active tDCS group, while patients with an anxiety diagnosis were overrepresented in the sham group. Because prefrontal tDCS outcomes may depend on gender (Dedoncker, Brunoni, Baeken, & Vanderhasselt, 2016), we repeated analyses without the female participants. This did not significantly change results.

**Table 1.** Demographical and clinical participant characteristics with mean and standard deviation values or count

		Active tDCS (*n* *=* 47)	Sham (*n* *=* 49)
Gender	Male:	41	48
	Female:	6	1
Age (years)^a^		40.5 (10.6)	44.4 (9.4)
Education level^b^	Low:	4	1
	Moderate:	30	30
	High:	13	18
Military status	Active-duty:	29	40
	Post-active veteran:	18	9
Number of deployments		2.6 (2.6)	3.3 (2.0)
Years since last deployment (years)		12.9 (11.3)	12.8 (10.0)
Treatment type during tDCS intervention^c^	EMDR:	8	8
	CBT:	22	26
	Other:	17	15
Use of psychoactive medication^d^	Yes:	18	15
	No:	29	34
Childhood trauma (based on CTQ-SF cut-off scores for moderate to extreme childhood trauma)	Yes:	30	30
	No:	17	18
			*1 missing*
ADHD diagnosis	Yes:	7	6
	No:	40	43
Attentional impulsivity (BIS-11)		20.4 (3.5)	20.6 (3.5)
Motor impulsivity (BIS-11)		21.9 (3.6)	22.7 (3.4)
Non-planning impulsivity (BIS-11)		28.7 (4.4)	27.4 (4.7)
Diagnosis^e^:			
Impulsive aggression		23	22
Anxiety		16	24
PTSD		25	25

a Age was entered as a covariate in the statistical analyses. Excluding the *Age* covariate from the models did not significantly change the results.

b Education level: low = high school education only, moderate = vocational degree, high = higher education degree.

c EMDR, eye movement desensitization and reprocessing therapy; CBT, cognitive behavioral therapy. Other treatments included: aggression regulation training, mindfulness-based therapy, couples therapy, maintenance therapy by social workers, and pharmacological treatment.

d The majority of psychoactive drugs used in our sample comprised selective serotonin or serotonin-norepinephrine reuptake inhibitors (SSRI's and SNRI's), benzodiazepines, atypical antipsychotic drugs, norepinephrine-dopamine reuptake inhibitors (NDRI's), and anticonvulsants. Analysis of the primary outcome measure (SST training scores) showed similar results across medicated and unmedicated patients. Also, excluding *Use of psychoactive medication* (*yes/no*) as a covariate from the models did not significantly change the results of any other measure.

e While most participants fulfilled criteria for either PTSD or anxiety or impulsive aggression, some participants fulfilled criteria for multiple stress-related diagnoses: PTSD and anxiety (*n* = 10), PTSD and impulsive aggression (*n* = 14), anxiety and impulsive aggression (*n* = 6), or all three diagnoses (*n* = 5).

### Safety

The intervention was well tolerated and no serious adverse events were reported. The only tDCS-related side effects were mild itching and burning sensations on the scalp (mean severity scores ± s.d. itching *–* active tDCS: 1.7 ± 0.7 *v.* sham: 1.4 ± 0.6; burning – active tDCS: 1.6 ± 0.7 *v.* sham: 1.3 ± 0.6; *p*'s < 0.001), and some tDCS participants noticed light skin redness that was absent in the sham group (active tDCS: 1.1 ± 0.6 *v.* sham: 1.0 ± 0.1; *p* = 0.010). Emotional state fluctuations during tDCS sessions were negligible and did not significantly differ between stimulation groups (mean STAI-6 item absolute change score: 0.26 ± 0.48; effects of *Stimulation group* and *Stimulation group* *×* *STAI-6 item* on change scores: *p*'s > 0.18).

### Primary outcome: inhibitory control training on the stop-signal task

Three participants showed very slow response times on session 1, preventing reliable SSRT computations. As this comprised <5% of the data, the a priori defined analyses were performed on the remaining sample (46 tDCS and 47 sham) (Jakobsen, Gluud, Wetterslev, & Winkel, 2017). A mean stop-signal response accuracy of 51.5% ± 7% confirmed successful stop-signal delay titration.

**Fig. 2.**
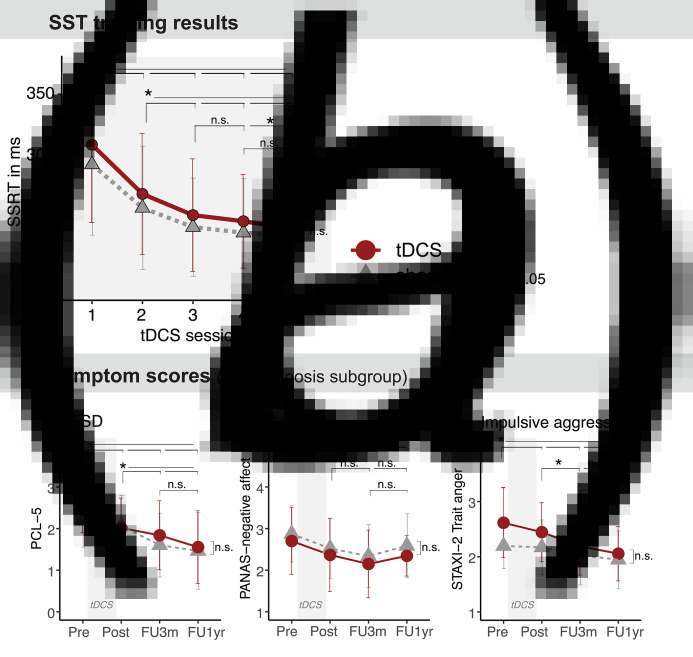
Mean SSRT **(***a*) and mean item scores on symptom scales **(***b***)** ± s.d. per stimulation group. n.s., non-significant. Please note that symptom scales were analyzed per subgroup of patients with the corresponding diagnosis, and that drop-out at follow-up reduced the sample sizes for FU-3m and FU-1yr assessments, see also Table 2.

The active *v.* sham tDCS groups did not significantly differ in overall SSRT scores or in SSRT improvement over sessions, as indicated by the non-significant effects of *Stimulation group* and the *Stimulation group* *×* *Time* interaction (respectively: *p* = 0.239, *η*^2^**_G_** = 0.011; *p* = 0.582, *η*^2^_G_ = 0.002). Only the main effect of *Time* was significant (*p* < 0.001, *η*^2^_G_ = 0.019). SSRT changes between sessions were tested with post-hoc Bonferroni-corrected pairwise *t* tests; the SSRT significantly decreased from session 1 to session 2 and all following sessions, from session 2 to session 3 and all following sessions, and from session 3 to session 5 (*p*'s < 0.01), see Fig. 2. When *Diagnosis* was entered as an additional between-subjects factor to explore possible differences between patient subgroups, the tDCS related effects remained non-significant (*Stimulation group*: *p* = 0.255, *η*^2^_G_ = 0.011; *Stimulation group* *×* *Time*: *p* = 0.905, *η*^2^_G_ < 0.001; *Stimulation group* *×* *Time* *×* *Diagnosis*: *p* = 0.201, *η*^2^_G_ = 0.009). However, beside a main effect of *Time* (*p* < 0.001, *η*^2^_G_ = 0.018), a significant *Time* *×* *Diagnosis* interaction appeared (*p* = 0.005, *η*^2^_G_ = 0.020). Based on visual inspection of the SSRTs per subgroup, the interaction seemed to reflect a relatively strong SSRT decrease in the PTSD subgroup compared to the anxiety and aggression subgroups (see online Supplementary Fig. S2). Next, despite the underpowered 2 × 5 mixed design for the diagnosis subgroups, the subgroups were analyzed separately. The main effect of *Time* remained significant among PTSD patients (*p* = 0.014, *η*^2^_G_ = 0.028), and was non-significant in the anxiety and aggression subgroups (respectively: *p* = 0.094, *η*^2^_G_ = 0.019; *p* = 0.083, *η*^2^_G_ = 0.036).

**Table 2.** Statistical outcomes of non-trained inhibitory control and symptom measures

		Go/no-go task – Go trial RT (in ms)		*Go/no-go task – total No-go trial error rate*		*D600 IAT effect*	
**tDCS**	**Pre** Noface: Neutral: Angry:	415 ± 38 392 ± 37 395 ± 35	*Group × Time:* *p* = .797 *η*^2^ _G_ < .001 *Group × Time × Distractor:* *p* = .310 *η*^2^ _G_ *< .001*	0.38 0.59 0.64	*Group × Time:* *p* = .727 *β* = 0.17 SE = 0.20 *Group × Time × Distractor:* *p* = .791 *β* = −0.02 SE = 0.24	0.67 ± .38	*Group × Time:* *p* = .140 *η*^2^ _G_ = .011
	**Post** Noface: Neutral: Angry:	409 ± 43 391 ± 42 392 ± 42		0.37 0.78 0.81		0.84 ± .27	
**Sham**	**Pre** Noface: Neutral: Angry:	403 ± 41 391 ± 43 389 ± 41		0.42 0.66 0.55		0.74 ± .30	
	**Post** Noface: Neutral: Angry:	397 ± 44 384 ± 44 387 ± 47		0.35 0.57 0.53		0.77 ± .38	

		*PTSD symptoms* (*PCL-5*)		*Anxiety symptoms* (*PANAS Negative Affect*)		*Aggression symptoms* (*STAXI-2 Trait Anger*)	
		*Mean per item*		*Mean per item*		*Mean per item*	
**tDCS**	**Pre**	2.45 ± .55 *n = 25*	*Group × Time* Pre-Post*:* *p* = .023 *η*^2^_G_ *= .010* *Group × Time* incl. FU's: *p* = .572 *η*^2^_G_ *= .*004	2.70 ± .80 *n = 16*	*Group × Time* Pre-Post: *p* = .843 *η*^2^ _G_ *< .001* *Group × Time* incl. FU's: p = .953 *η*^2^_G_ = .001	2.60 ± .61 *n = 23*	*Group × Time* Pre-Post: *p* = .243. *η*^2^_G_ *= .005* *Group × Time* incl. FU's: *p* = .980 *η*^2^ _G_ < .001
	**Post**	2.02 ± .72 *n = 23*		2.37 ± .88 *n = 15*		2.37 ± .58 *n = 22*	
	**FU3m**	1.84 ± .83 *n = 22*		2.15 ± .82 *n = 14*		2.13 ± .57 *n = 20*	
	**FU1yr**	1.55 ± .87 *n = 13*		2.34 ± .49 *n = 9*		1.99 ± .51 *n = 10*	
**Sham**	**Pre**	2.19 ± .63 *n = 25*		2.90 ± .68 *n = 24*		2.28 ± .58 *n = 22*	
	**Post**	2.07 ± .72 *n = 23*		2.56 ± .72 *n = 22*		2.28 ± .64 *n = 20*	
	**FU3m**	1.60 ± .77 *n = 21*		2.36 ± .74 *n = 19*		2.08 ± .56 *n = 17*	
	**FU1yr**	1.46 ± .92 *n = 18*		2.56 ± .75 *n = 16*		1.98 ± .56 *n = 12*	

		*Depressive symptoms* (*BDI-II*)		*General wellbeing* (*OQ45*)				
		*Mean per item:*		Symptom Distress	Inter-personal Relations	Social Role	Anxious Arousal	
**tDCS**	**Pre**	1.93 ± .49 *n = 47*	*Group × Time* Pre-Post: *p = .885* *η*^2^ _G_ *< .001* *Group × Time* incl. FU's: *p = .213* *η*^2^_G_ *= .007*	1.70 ± .62 *n = 47*	1.42 ± .57	1.63 ± .60	1.75 ± .70	*Group × Subscale × Time* Pre-Post: *p* = .266 *η*^2^ _G_ < .001 *Group × Subscale × Time* incl. FU's: *p* = .374 *η*^2^_G_ = .001
	**Post**	1.71 ± .51 *n = 44*		1.49 ± .65 *n = 44*	1.28 ± .59	1.49 ± .56	1.50 ± .72	
	**FU3m**	1.65 ± .56 *n = 40*		1.49 ± .68 *n = 41*	1.29 ± .60	1.43 ± .56	1.58 ± .72	
	**FU1yr**	1.62 ± .48 *n = 25*		1.27 ± .60 *n = 25*	1.13 ± .57	1.37 ± .51	1.34 ± .68	
**Sham**	**Pre**	2.03 ± .47 *n = 49*		1.76 ± .52 *n = 49*	1.57 ± .60	1.43 ± .56	1.77 ± .53	
	**Post**	1.83 ± .49 *n = 46*		1.62 ± .51 *n = 45*	1.43 ± .62	1.35 ± .53	1.62 ± .51	
	**FU3m**	1.69 ± .41 *n = 41*		1.49 ± .60 *n = 41*	1.34 ± .65	1.27 ± .39	1.48 ± .59	
	**FU1yr**	1.74 ± .57 *n = 33*		1.43 ± .68 *n = 31*	1.37 ± .66	1.28 ± .46	1.41 ± .68	

FU3m = 3-months follow-up assessment. FU1yr = 1-year follow-up assessment.

Concerning the no-signal RT, no significant effects of active *v.* sham tDCS appeared either (*Stimulation group* main effect: *p* = 0.338, *η*^2^_G_ = 0.012; *Stimulation group* *×* *Time* interaction: *p* = 0.309, *η*^2^_G_ = 0.003), although participants did become faster over sessions (main effect of *Time*: *p* < 0.001, *η*^2^_G_ = 0.024). For further details on the no-signal RT, see online Supplementary Fig. S2.

In an additional analysis, we explored if tDCS effects on inhibitory control training would depend on baseline levels of inhibitory control, which was assessed by the go/no-go task. To that end, we regressed the total SSRT improvement from sessions 1 to 5 on the predictors *pre-assessment Go/no-go scores* (RT and accuracy) and *Stimulation group*. Results showed no evidence for a dependence of tDCS effects on baseline inhibitory control performance (*Stimulation group* *×* *Go/NoGo scores* interaction effects: *p*'s > 0.418). Analysis details can be found in the online Supplementary materials.

### Secondary outcomes of inhibitory control

Means and standard deviations per group are reported in Table 2, together with the outcomes of the *Stimulation group* *×* *Time* interaction effects of interest.

#### Go/no-go task

Go/no-go data from 80 participants were available for analysis (40 tDCS, 40 sham; missings due to insufficient (<100) completed trials, *n* = 5; post-assessment unavailable or completed >1 week after tDCS intervention, *n* = 11). TDCS did not influence response speed or response inhibition accuracy: pre-to-post intervention changes in RT or no-go accuracy were not significantly different between active and sham tDCS groups (see Table 2). Response speed did not significantly change over time or differ between groups at all (main effect *Time*: *p* = 0.273, *Stimulation group*: *p* = 0.374). For accuracy, a significant *Go/no-go* *×* *Time* interaction (*p* = 0.005; *β* = 0.41, std. error = 0.15) and a significant *Stimulation group* *×* *Time* interaction appeared (*p* = 0.008; *β* = −0.17, std. error = 0.06). Bonferroni-corrected pairwise *t* tests showed that go-trial accuracy increased from pre- to post-assessment in both stimulation groups (go-trials – pre *v.* post: *p* < 0.001). Such effects were not found for no-go accuracy (i.e. response inhibition accuracy – pre *v.* post: *p* > 0.999). Moreover, the stimulation groups differed in overall performance accuracy at post-assessment, where the sham group made significantly less errors than the active tDCS group (pre-assessment – active tDCS *v.* sham: *p* = 0.898; post-assessment – active tDCS *v.* sham: *p* = 0.011), suggesting a lack of improvement in overall performance accuracy over time in the active tDCS group. Again, no group differences were found specifically in no-go accuracy (response inhibition). Furthermore, the face distractors significantly impaired task performance: *Distractor condition* showed a significant main effect on both RT and accuracy (*p*'s < 0.001). Follow-up *t* tests and χ^2^ tests showed that RTs were faster on trials with face distractors (distractor *v.* no-distractor: *p* < 0.001, neutral *v.* angry distractor: *p* = 0.690). This distractor-induced RT acceleration also yielded a *Stimulation group* *×* *Distractor condition* interaction (*p* = 0.047), showing it was more pronounced in the active *v.* sham tDCS group (*p* = 0.034). Error rates increased from no-distractor- to neutral face distractor- to angry face distractor-trials (*p*'s < 0.045).

#### IAT

IAT data from 84 participants were available for analysis (43 tDCS, 41 sham; missings due to post-assessment unavailable or completed >1 week after tDCS intervention, *n* = 12). Pre-to-post intervention changes in the D600 IAT effect did not significantly differ between the active tDCS and sham group (see Table 2). The IAT effect significantly increased from pre- to post-assessment (*p* = 0.042, *η*^2^_G_ = 0.021), indicating a possible reduction in inhibitory control over biases due to automatic associations. The Quad model ‘overcoming bias’ parameter did not appear significantly affected by *Stimulation group*, but the overall model fit was very low suggesting the Quad model results were not reliable (model fit for post-assessment IAT data – tDCS group: G^2^(6) = 11.33, *p* = 0.079, AIC = 23.33; sham: group G^2^(6) = 13.00, *p* = 0.043, AIC = 25.00). The full analysis is reported in the online Supplementary materials.

### Symptoms

The analysis of PTSD symptoms was only carried out within the subgroup of PTSD patients, the analysis on anxiety symptoms only within the subgroup of anxiety patients, and likewise for the impulsive aggression patients. Data were available for analysis per diagnosis subgroup as indicated in Table 2 (missings due to unavailable post-assessment or completed >1 week after tDCS intervention: PTSD: *n* = 5; anxiety: *n* = 2; aggression: *n* = 3). Beside an overall significant reduction in symptom levels over time (main effect of *Time*: *p*'s < 0.001, *η*^2^_G_'s > 0.008), the active tDCS *v.* sham groups did not significantly differ in symptom levels reductions, except for a slightly stronger reduction in PCL-5 scores in the active tDCS *v.* sham group due to higher baseline PTSD symptoms levels in the active tDCS group (see Table 2 and Fig. 2). When the 3-months and 1-year follow-ups were taken into account, these results did not substantively change, see Table 2. PANAS Positive Affect and STAXI-2 Anger Expression and Control scales did not show significant effects of tDCS *v.* sham (statistical results are reported in the online Supplementary material).

### Exploratory analyses on the relation between inhibitory control and symptom severity

At baseline, higher symptom severity on all scales significantly correlated with worse stop-signal task inhibitory control performance, see Table 3. Baseline no-go-accuracy significantly correlated with PCL-5 and BDI-II scores. No other baseline inhibitory control measure correlated significantly with symptom levels.

**Table 3.** Correlation matrix with baseline measures of symptom severity and inhibitory control.

	1	2	3	4	5	6	7
1. PCL-5							
2. PANAS negative affect	0.72**						
3. STAXI-2 trait anger	0.31*	0.33*					
4. BDI-II	0.74**	0.69**	0.22*				
5. SSRT (*reversed*)	−0.50**	−0.33*	−0.25*	−0.36**			
6. Go/no-go: RT	−0.04	0.08	−0.04	−0.01	0.04		
7. Go/no-go: no-go accuracy	−0.27*	−0.18	−0.01	−0.31*	0.43**	0.15	
8. IAT effect (*reversed*)	0.15	0.16	0.01	0.20	−0.01	0.16	0.06

Higher symptom scores reflect higher symptom severity, lower (reversed) inhibitory control scores reflect worse inhibitory control performance. Note that the SSRT used for the baseline correlations was measured during the first tDCS session.

* *p* < 0.05, **p < 0.001.

The overall improvement in SSRT or the achieved level of SSRT on session 5 did not significantly predict symptom reductions (all *p*'s > 0.28, full statistical outcomes are reported in the online Supplementary material). These results suggest no link between short-term inhibitory control improvements and symptom relief.

## Discussion

Inhibitory control is thought to play a role in symptoms of PTSD, anxiety, and impulsive aggression. Here, the effects of a tDCS-combined inhibitory control training on pre–post measures of inhibitory control and symptoms were for the first time investigated in a preregistered RCT with a large clinical sample of military patients with these stress-related disorders. Contrary to previous findings (Ditye et al., 2012), we failed to find an effect of anodal tDCS over the right IFG *v.* sham on performance during the stop-signal task inhibitory control training. No support was found either for tDCS effects on post-intervention non-trained inhibitory control nor on symptom levels of PTSD, anxiety, or impulsive aggression. Hence, despite positive effects of tDCS on inhibitory control in healthy individuals (Mayer et al., 2020) and on symptoms of PTSD and anxiety in patients (Ahmadizadeh, Rezaei, & Fitzgerald, 2019; van ’t Wout-Frank, Shea, Larson, Greenberg, & Philip, 2019; Vicario, Salehinejad, Felmingham, Martino, & Nitsche, 2019), we found no evidence to support that right IFG tDCS combined with inhibitory control training with our experimental set-up can effectively improve inhibitory control or stress-related symptoms in these patients. These results raise questions on why the tDCS effects on inhibitory control did not replicate in our clinical sample, and, subsequently, what may be more effective ways to modulate clinically relevant cognitive processes and stress-related symptoms with non-invasive brain stimulation.

### Effects of tDCS-combined training on inhibitory control

A substantial body of single-session tDCS research (Mayer et al., 2020; Schroeder et al., 2020) and a multiple-session tDCS-training intervention study (Ditye et al., 2012) in healthy participants showed successful improvements in inhibitory control performance with tDCS settings not so different from ours (current intensity: 1–1.5 mA; anode over the right IFG; cathode on left orbital area or left cheek; duration: 10–30 min). Compared to the study of Ditye and coworkers, we extended the training and stimulation duration per session. Yet, the effects of tDCS were not replicated. Perhaps by using a current density on the low end (0.036 mA/cm^2^) of the range used for successful tDCS-enhanced stop-signal task performance in other studies (0.028–0.125 mA/cm^2^) (Mayer et al., 2020), the induced electrical field was too weak to modulate right IFG activity to an extent that would produce measurable behavioral changes [see, e.g. Li et al. (2019)]. On the other hand, higher current densities do not necessarily follow a linear increase of tDCS effectivity (Yavari et al., 2018).

Secondly, although we used a montage as applied by other studies stimulating the IFG, there is uncertainty about the anode placement relative to the IFG. Simulations of the electrical field on one example brain showed a peak intensity located slightly above the IFG (see online Supplementary Fig. S1). Although inconclusive, the target region may have received suboptimal stimulation. To more effectively target inhibitory control, the anode could be placed somewhat lower to better focus the electrical field on the right IFG, e.g. on 10-20 system EEG positions F8 or F10 (Coffman et al., 2012; Schroeder et al., 2020), or higher, e.g. on position F4 to focus the field on the dorsolateral PFC (Dousset et al., 2020; Salehinejad, Wischnewski, Nejati, Vicario, & Nitsche, 2019). However, tDCS with the anode placed on the F8-Cz Fz-T4 crossing, as in our study, has also shown successful response inhibition enhancement (Mayer et al., 2020; Schroeder et al., 2020). Technical tDCS parameter settings therefore do not seem to fully explain our null results.

Alternatively, we possibly over-trained a relatively simple inhibitory control task. As the primary physiological effects of tDCS act upon ongoing neuronal and synaptic activity (Kronberg, Bridi, Abel, Bikson, & Parra, 2017; Liebetanz, 2002; Nitsche & Paulus, 2000), tDCS appears suitable to enhance processes that depend on synaptic plasticity, like learning and memory processes. Correspondingly, in Ditye's study (Ditye et al., 2012), tDCS seemed to act as a necessary condition for an inhibitory control learning effect to occur. However, our extended training sessions produced clear learning curves in both stimulation groups, and we found no support for baseline inhibitory control performance to predict tDCS effectivity. Together with indications that tDCS-enhancement can supersede after experience-dependent learning [see, e.g. Fehring et al. (2019)], this suggests that tDCS might have had little opportunity to further enhance training processes in our study. Moreover, patients with stress-related disorders may specifically show impulsivity in the emotional domain (Johnson, Carver, & Joormann, 2013), and tDCS effects on cognitive and emotional outcomes seem to depend on active emotion regulation, cognitive effort and neural activity in the targeted area (Gill, Shah-basak, & Hamilton, 2015; Nord et al., 2019; Smits et al., 2020). Our response inhibition training may have failed to adequately incorporate these factors due to its non-emotional nature and low cognitive load. Also, non-trained inhibitory control tasks (go/no-go task and IAT) showed no evidence for tDCS effects, in line with expectations that effects do not transfer in the absence of tDCS effects on trained tasks (Berryhill & Martin, 2018). Altogether, conditions for tDCS efficacy in these patients may crucially include emotionally challenging tasks during stimulation.

### Effects of tDCS-combined training on symptoms

In light of the null-effects on inhibitory control, the tDCS intervention would not affect symptom levels of PTSD, anxiety, and aggression via such mediating cognitive processes. On the other hand, tDCS effects on symptoms without concurrent cognitive improvement have previously been shown in depression (Martin et al., 2018) and PTSD patients (Ahmadizadeh et al., 2019), suggesting that prefrontal tDCS may also affect symptoms via other mechanisms. However, on stress-related as well as mood symptoms and general mental well-being, no evidence for tDCS effects was found. Possibly, such non-specific tDCS effects require more sessions and a shorter between-session-interval (max. 1 day) (Alonzo, Brassil, Taylor, Martin, & Loo, 2012). Patients in both stimulation groups did show significant symptom reduction over the course of the intervention, presumably as a result primarily of ongoing therapeutic processes of regular treatment.

### Future directions

To find more effective ways to target stress-related symptoms with tDCS, the next steps should be to identify what are the relevant brain processes that facilitate recovery, and to determine under what conditions tDCS effectively modulates those brain processes. Brain state may constitute one of the most important but also unresolved factors of influence on tDCS effectivity. Whereas we intended to attune brain states during the intervention across participants by applying a concurrent cognitive task, the combination with neuroimaging methods can help to better study brain state in parallel to the behavioral and clinical effects of tDCS [see, e.g. Nord et al. (2019)]. Regarding inhibitory control as a cognitive target, exploratory analyses confirmed the association with stress-related symptoms, but not with symptom relief. An alternative target may be tDCS over the dorsolateral PFC (Brunoni & Vanderhasselt, 2014) to modulate working memory deficits in stress-related disorders [see e.g. Scott et al. (2015)] which can contribute to symptom relief (Schweizer et al., 2017). Successful attempts to enhance effects of cognitive behavioral or exposure psychotherapy with prefrontal stimulation (Herrmann et al., 2017; Nord et al., 2019; van ’t Wout-Frank et al., 2019) also suggest that tDCS interventions might be further developed in existing clinical applications. More placebo-controlled clinical trials are encouraged to examine whether this is a viable option.

### Limitations

Limitations in our study may restrict the generalization of our results. First, pre- and post-intervention measures were assessed online. As a trade-off for a lower travel burden for patients (Smits, de Kort, & Geuze, 2021), this could have reduced the measurement sensitivity to detect (possibly weak) tDCS effects. On the other hand, cognitive assessment through online experiments appear reliable (Gladwin & Vink, 2020). Also, we carried out this study in an (ex-)military, predominantly male sample. Excluding data from female participants did not essentially change the results, and our sample represented a broad and heterogeneous group, but military personnel in general may represent a relatively homogenous population due to rigid selection and training procedures. Our outcomes may therefore not directly translate to other populations.

## Conclusion

The current RCT in military patients with stress-related symptoms provides no evidence for short-term or long-term benefits of 5 sessions of 20-min tDCS targeting the right IFG at an intensity of 1.25 mA combined with response inhibition training, on inhibitory control or PTSD, anxiety, and impulsive aggression symptoms. For these patients, tDCS may be more effective in higher doses (e.g. higher current density, more sessions) or when combined with emotionally challenging tasks or psychotherapy. Gaining insight in determinants of tDCS efficacy and convenient brain targets for neuromodulation in stress-related disorders will allow the tailoring of future tDCS interventions.

## Data Availability

The data that support the findings of this study are available from the corresponding author, FS, upon reasonable request.

## Supplementary material

For supplementary material accompanying this paper visit https://doi.org/10.1017/S0033291721000817. click here to view supplementary material
