# Supplementary material for: Effects of tDCS during inhibitory control training on performance and PTSD, aggression and anxiety symptoms: a randomized-controlled trial in a military sample
Source: Psychol Med. 2021 Mar 24;52(16):3964–74. doi: 10.1017/S0033291721000817 (PMC9811348; doi:10.1017/S0033291721000817)
Supplement: Supplementary file 1 [file S0033291721000817sup001.pdf]

SUPPLEMENTARY MATERIALS

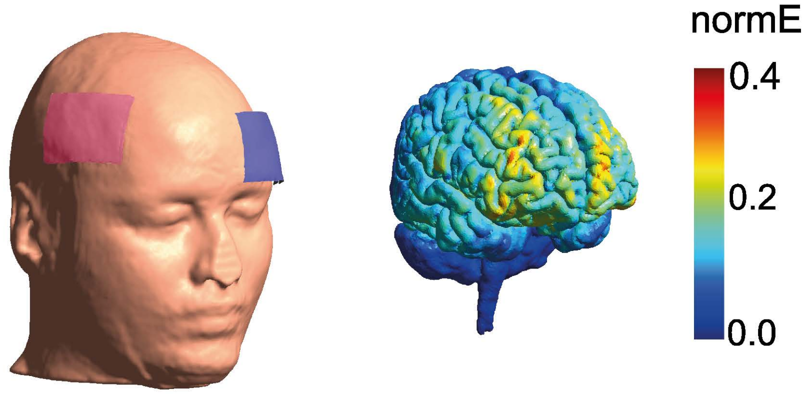

Figure S.1. A simulated induced electrical field image with the applied tDCS montage, created with SimNIBS 2.1 (Thielscher et al., 2015).

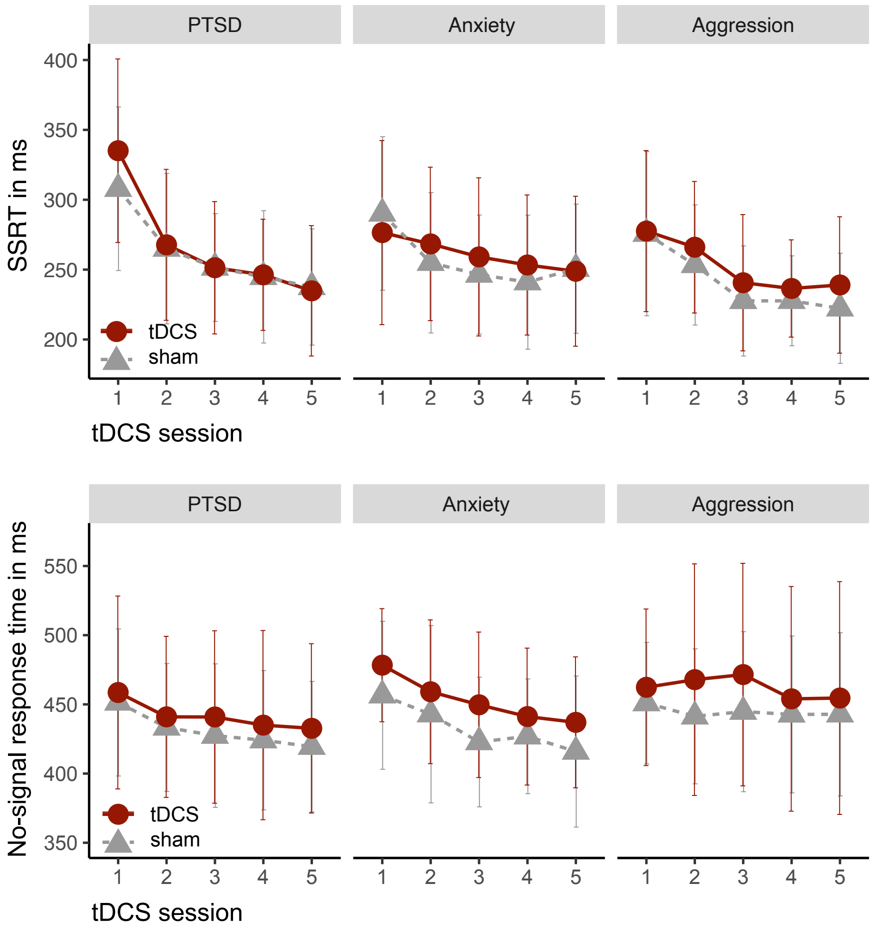

Figure S.2. Mean SSRT (above) and no-signal RT (below) ± SD per *Stimulation group* for each diagnosis subgroup. No-signal RT analysis results: When *Diagnosis* was entered into the model, the main and interaction effects of *Stimulation group* did not significantly change (p's > .180, η^2_G's < .018). In line with the SSRT outcomes, a significant *Time* × *Diagnosis* interaction appeared (p = .033, η^2_G = .007). In separate analyses of each diagnosis subgroup, PTSD and anxiety patients showed a significant main effect of *Time* (p's < .001, η^2_G's > .030), corresponding to decreasing RTs over sessions. No *Time* effect was observed in the impulsive aggression subgroup (p = .429, η^2_G = .004).

Influence of baseline inhibitory control on tDCS effects in SST training

The SSRT change score (ΔSSRT = SSRT at session 5 – SSRT at session 1) was regressed on *pre-assessment Go/no-go scores* (RT and accuracy, separately) together with the predictor *Stimulation group,* and their interaction. The results are presented in Table S.1. *Go-RT* and *No-go accuracy* did not interact significantly with *Stimulation group* in predicting SSRT change, suggesting that baseline go/no-go performance did not influence tDCS effects on SSRT enhancement. No-go accuracy did have a main effect on the SSRT change score. Correlation analysis showed that lower no-go accuracy at baseline was associated with stronger SSRT improvements during training (r = .24, p = .036), implying that worse inhibitory control performance at baseline may leave more room for performance improvement during inhibitory control training.

Table S.1. Regression outcomes of models testing the predictive value of baseline go/no-go performance.

Predictor	*F* (df)	*p*-value
***Formula**: ΔSSRT ~ Stimulation group * Go-RT at pre-assessment*		
Stimulation group	0.25 (1)	.619
Go-RT	0.03 (1)	.854
Stimulation group × Go-RT	0.66 (1)	.418
***Formula**: ΔSSRT ~ Stimulation group * No-go accuracy at pre-assessment*		
Stimulation group	0.26 (1)	.610
No-go accuracy	4.69 (1)	.033
Stimulation group × No-go accuracy	0.01 (1)	.933

IAT – Quad model details and outcomes

The Quad model (Conrey et al., 2005) includes the following components that drive response behavior in the IAT: association activation (AC: “the likelihood that automatic bias is activated by a stimulus”), discriminability (D: “the likelihood that a correct response can be determined”), overcoming bias (OB: “the likelihood that automatic bias is overcome”), and guessing (G: “the likelihood that, in the absence of other information, a guessing bias drives responses). The D and G parameters were defined for target words and attribute words separately (i.e., for target words: D_{target} and G_{target}, and for attribute words: $D_{attribute}$ and $G_{attribute}$). A single parameter was defined for AC and OB, as bidirectional

associations were assumed (Greenwald et al., 2002). The model was fitted on the number of correct and incorrect responses per trial category and task phase from all participants, separately for the pre- and post-assessment and for the active tDCS and sham groups.

To test group differences in the overcoming bias (OB) parameter at post-assessment, we tested the free model (for parameter estimations, see Table S.2.) against a model where OB was constrained to $OB_{tDCS} = OB_{sham}$ in a loglikelihood ratio test. The model fit improved very little (ΔAIC = -1.09), indicating that the OB parameter did not differ significantly between groups post-intervention. To test changes in OB over time in both groups, we tested the free model against a model where OB was constrained to $OB_{Pre} = OB_{Post}$. This slightly reduced model fit in both stimulation groups as reflected by small increases in Akaike's Information Criterion (ΔAIC) (tDCS group: ΔAIC = +3.83, sham group: ΔAIC = +0.54), indicating that OB changed from pre-to-post intervention. OB decreased over time, representing reduced implicit inhibitory control, see Table S.2.

Table S.2. Quad model parameters for latent variables underlying IAT performance.

	**tDCS**				**Sham**			
	Pre-assessment		***Post-assessment***		***Pre-assessment***		***Post-assessment***	
Parameter	*Estimate*	*CI -95%*	*Estimate*	*CI -95%*	*Estimate*	*CI -95%*	*Estimate*	*CI -95%*
AC	.41	[.17, .64]	.11	[-.12, .33]	.14	[-.16, .45]	.13	[-.10, .37]
D_{target}	.94	[.92, .95]	.91	[.89, .93]	.95	[.94, .97]	.91	[.89, .93]
$D_{attribute}$	.96	[.95, .98]	.93	[.91, .94]	.95	[.93, .97]	.94	[.92, .96]
G_{target}	.52	[.42, .63]	.50	[.42, .57]	.54	[.42, .65]	.47	[.39, .55]
$G_{attribute}$	.61	[.47, .75]	.64	[.56, .73]	.44	[.32, .55]	.50	[.40, .61]
OB	1.00	[.96, 1.04]	.69	[-.17, 1.52]	.84	[.39, 1.28]	.58	[-.30, 1.45]

Dot-probe task

The dot-probe task measures attentional biases for threat. In this task, a pair of face cues (one angry face and one neutral face) were presented on a computer screen, divided over the upper and lower half of the screen. After a variable cue-stimulus interval (CSI; 200, 400, 600, 900 or 1200 ms), the face cues were replaced by a probe stimulus ('>>>' or '<<<') and a distractor stimulus ('\/\/' or '/\/\'). Participants were instructed to identify the direction of the probe stimulus (left or right) as fast as possible by pressing the correct button on the keyboard: 'F' or 'J'. The probe stimulus randomly

appeared in the angry face cue location or in the neutral face cue location. An attentional bias toward the threat (here: angry face) location is induced by the fast attention capture of threat cues, especially at short CSIs (Cisler & Koster, 2010). A threat avoidance bias is also found in PTSD and anxiety patients, especially at longer CSIs. The attentional bias is computed as the RT difference between stimuli in the threat vs. neutral location (RT threat – RT neutral). The dot-probe task was only assessed post-intervention.

Results. Data were not available for 8 participants who did not complete the dot-probe task, leaving a sample of 88 for attention bias analysis (42 active tDCS, 46 sham). The attentional bias scores across CSI durations showed a very low split-half reliability of $r = -0.10$. This is not surprising in light of recent insights: dot-probe performance does often not reliably measure attentional bias (McNally, 2019), although this task has also shown reliable results (see e.g. [Gladwin & Vink, 2020]). Considering that the tDCS intervention could have modulated aspects of dot-probe task performance, we carried out the preregistered analysis in spite of the low reliability.

Overall, both active tDCS and sham groups showed very small attentional bias scores that did not significantly differ from zero (attentional bias score in ms – active tDCS: M = 5.3 ±27.7; sham: -1.4 ±25.1). Regardless of controlling for *Age* and *Medication use*, the attentional bias scores were not significantly influenced by *Stimulation group* or *CSI duration* (all p's > .38), see Table S.3.

Table S.3. Attentional bias scores (mean ±standard deviation) and analysis results

	CSI	Attentional bias score	Effect	*p*	η^2_G
**Active tDCS**	200 ms	-5.4 ±81.5	*Stimulation group*	.629	.001
	400 ms	-17.1 ±68.4	*CSI*	.386	.011
	600 ms	17.2 ±87.6	*Stimulation group × CSI*	.756	.005
	900 ms	-1.5 ±70.2			
	1200 ms	-8.1 ±73.0			
**Sham**	200 ms	-0.3 ±84.8			
	400 ms	-10.4 ±70.4			
	600 ms	11.9 ±72.2			
	900 ms	1.7 ±80.1			
	1200 ms	1.7 ±73.1			

PANAS Positive Affect and STAXI-2 Anger Control subscales results

PANAS Positive Affect. There were no significant main effects of *Stimulation group* or *Time* (p's > .19) on Positive Affect scores, and no significant *Stimulation group* × *Time* interaction effect ($p = .244$, $\eta^2_G = .006$).

STAXI-2 Anger Expression and Control. For the STAXI-2 Anger Expression scales, only the interaction *Time* × *Subscale* was significant ($p < .001$, $\eta^2_G = .019$). As expected, the Expression subscale scores decreased from pre- to post-assessment, indicating a reduction in anger expression (mean item scores – Anger Expression Out: M_{pre} ±SD = 2.5 ±0.5, M_{post} = 2.3 ±0.5; Anger Expression In: M_{pre} = 2.4 ±0.5, M_{post} = 2.4 ±0.5). Pairwise comparisons showed that the Express Anger Out subscale significantly decreased from pre- to post-assessment ($p = .018$), and from post-assessment to the 1-year follow-up ($p = .006$). The Express Anger In subscale did not significantly change between subsequent measurements (all p's > .120). The Control subscale scores increased significantly from pre- to post-assessment (respectively: $p = .021$, $p = .043$), indicating more anger control (Anger Control Out: M_{pre} ±SD = 2.1 ±0.6, M_{post} = 2.3 ±0.5; Anger Control In: M_{pre} = 2.2 ±0.6, M_{post} = 2.5 ±0.5), but showed no further change to the follow-ups at 3 months and 1 year (p's > .100). The interaction effects of interest, *Stimulation group* × *Time* ($p = .533$) and *Stimulation group* × *Time* × *Subscale* ($p = .743$) were not significant.

Exploratory analyses – Statistical outcomes of regression models

Table S.4. Regression outcomes of models testing the predictive value of SSRT improvement.

**Formula**: Δsymptom score ~ Stimulation group + ΔSSRT	Predictor	*F* (df)	*p*-value
Outcome: Δsymptom score PCL-5			
	Stimulation group	1.43 (1)	.235
	**ΔSSRT**	**0.30 (1)**	**.583**
Outcome: Δsymptom score PANAS Negative Affect			
	Stimulation group	0.01 (1)	.936
	**ΔSSRT**	**0.05 (1)**	**.818**
Outcome: Δsymptom score STAXI-2 Trait Anger			
	Stimulation group	2.48 (1)	.119
	**ΔSSRT**	**1.18 (1)**	**.280**

Table S.5. Regression outcomes of models testing the predictive value of achieved SSRT level on session 5.

Formula: Post-assessment symptom score ~ Stimulation group + pre-assessment symptom score + SSRT on session 5

Predictor	F (df)	p-value
Outcome: Post-assessment symptom score PCL-5		
Pre-assessment symptom score PCL-5	225.10 (1)	<.001
Stimulation group	2.27 (1)	.135
**SSRT on session 5**	**0.00 (1)**	**.980**
Outcome: Post-assessment symptom score PANAS Negative Affect		
Pre-assessment symptom score PANAS Negative Affect	120.09 (1)	<.001
Stimulation group	0.34 (1)	.562
**SSRT on session 5**	**0.17 (1)**	**.680**
Outcome: Post-assessment symptom score STAXI-2 Trait Anger		
Pre-assessment symptom score STAXI-2 Trait Anger	175.02	<.001
Stimulation group	0.09 (1)	.761
**SSRT on session 5**	**0.510 (1)**	**.477**

References

Cisler, J. M., & Koster, E. H. W. (2010). Mechanisms of attentional biases towards threat in anxiety disorders: An integrative review. In *Clinical Psychology Review*. https://doi.org/10.1016/j.cpr.2009.11.003

Conrey, F. R., Sherman, J. W., Gawronski, B., Hugenberg, K., & Groom, C. J. (2005). Separating multiple processes in implicit social cognition: the quad model of implicit task performance. *Journal of Personality and Social Psychology*, *89*(4), 469–487. https://doi.org/10.1037/0022-3514.89.4.469

Gladwin, T. E., & Vink, M. (2020). Spatial anticipatory attentional bias for threat: Reliable individual differences with RT-based online measurement. *Consciousness and Cognition*, *81*, 102930. https://doi.org/10.1016/j.concog.2020.102930

Greenwald, A. G., Rudman, L. A., Nosek, B. A., Banaji, M. R., Farnham, S. D., & Mellott, D. S. (2002). A unified theory of implicit attitudes, stereotypes, self-esteem, and self-concept. *Psychological Review*. https://doi.org/10.1037/0033-295X.109.1.3

McNally, R. J. (2019). Attentional bias for threat: Crisis or opportunity? In *Clinical Psychology Review*. https://doi.org/10.1016/j.cpr.2018.05.005

Thielscher, A., Antunes, A., & Saturnino, G. B. (2015). Field modeling for transcranial magnetic

stimulation: A useful tool to understand the physiological effects of TMS? *2015 37th Annual International Conference of the IEEE Engineering in Medicine and Biology Society (EMBC)*, 222–225. https://doi.org/10.1109/EMBC.2015.7318340
